# Effect of Low-Dose Aspirin on Soluble FMS-Like Tyrosine Kinase 1/Placental Growth Factor (sFlt-1/PlGF Ratio) in Pregnancies at High Risk for the Development of Preeclampsia

**DOI:** 10.3390/jcm8091429

**Published:** 2019-09-10

**Authors:** Karoline Mayer-Pickel, Vassiliki Kolovetsiou-Kreiner, Christina Stern, Julia Münzker, Katharina Eberhard, Slave Trajanoski, Ioana-Claudia Lakovschek, Daniela Ulrich, Bence Csapo, Uwe Lang, Barbara Obermayer-Pietsch, Mila Cervar-Zivkovic

**Affiliations:** 1 Department of Obstetrics and Gynecology, Division of Obstetrics, Medical University of Graz, A-8036 Graz, Austria; 2 Department of Internal Medicine, Div. of Endocrinology and Diabetology, Endocrinology Lab Platform, Medical University of Graz, A-8036 Graz, Austria; 3 Department for Core Facility Computational Bioanalytics, Center for Medical Research (ZMF), Medical University of Graz, A-8036 Graz, Austria

**Keywords:** adverse pregnancy outcome, aspirin, first trimester screening for preeclampsia, high-risk pregnancies, preeclampsia, sFlt1/PlGF ratio

## Abstract

Background: Soluble FMS-like Tyrosine Kinase 1 (sFlt-1) and placental growth factor (PlGF) have been reported to be highly predictive several weeks before the onset of preeclampsia. Objective: To investigate longitudinal changes of serum levels sFlt-1 and PlGF in pregnant women at high risk for the development of preeclampsia and to reveal an impact of aspirin on maternal serum concentrations of sFlt-1 and PlGF. Methods: This was a prospective longitudinal study in 394 women with various risk factors for the development of preeclampsia (chronic hypertension, antiphospholipid syndrome/APS or systemic lupus erythematosus/SLE, thrombophilia, women with a history of preeclampsia, pathologic first trimester screening for preeclampsia) and 68 healthy women. Serum levels of sFlt-1 and PlGF were measured prospectively at 4-week intervals (from gestational weeks 12 until postpartum). Results: The sFlt-1/PlGF ratio was significantly higher in women with an adverse obstetric outcome compared to women with a normal pregnancy, starting between 20 and 24 weeks of gestation. There was no effect of aspirin on sFlt-1/PlGF ratio in women with chronic hypertension, APS/SLE, thrombophilia and controls. The use of aspirin showed a trend towards an improvement of the sFlt-1/PlGF ratio in women with preeclampsia in a previous pregnancy and a significant effect on the sFlt-1/PlGF ratio in women with a pathologic first trimester screening for preeclampsia. Conclusions: Our findings reveal an impact of aspirin on sFlt-1/PlGF ratio in women with a pathologic first trimester screening for preeclampsia, strongly supporting its prophylactic use.

## 1. Introduction

Preeclampsia is a pregnancy-specific multiorgan disorder, complicating 3–5% of all pregnancies [1,2]. Despite advances in fetomaternal management, preeclampsia is still a major cause of maternal and neonatal morbidity and mortality worldwide, especially in developing countries. It is a known fact that the prevalence of preeclampsia is 3–5 times higher in women with preeclampsia in a previous pregnancy as well as in women with chronic hypertension [3,4]. Furthermore, pregnancies in women with certain autoimmune diseases such as systemic lupus erythematosus (SLE) or antiphospholipid syndrome (APS) are complicated by preeclampsia in up to 8–35% [5,6,7,8,9,10]. An association of maternal thrombophilia and preeclampsia has been reported, although with conflicting results [11,12]. Preeclampsia is not a single disease, but rather a syndrome, affecting many organs and is characterized by endothelial dysfunction. Although the exact mechanisms are still unknown, several systemic processes have been proposed and are well accepted: Angiogenic imbalance, oxidative stress, and exaggerated systemic inflammation [13,14,15,16]. Daily administration of low-dose aspirin (LDA) has a modest beneficial effect in reducing the rate of preterm preeclampsia [17,18,19,20,21,22]. It has been demonstrated that the use of LDA from 11 to 14 weeks of gestation until 36 weeks of gestation reduces the incidence of early onset preeclampsia by approximately 60% [21]. According to several previous studies, aspirin improves implantation and placentation and has vasodilatory effects by increasing prostacyclin production. Aspirin seems to have a direct effect on platelets and might improve endothelial dysfunction [23].

The effect of aspirin on angiogenic factors, such as soluble FMS-like Tyrosine Kinase 1 (sFlt-1) and placental growth factor (PlGF) is the subject of ongoing interest and has been examined in several in vitro studies.

The aim of the present study was to evaluate the serum levels of sFlt-1 and PlGF in women at high risk for the development of preeclampsia longitudinally during pregnancy and to determine a potential impact of LDA on the maternal serum concentrations of these angiogenic markers.

## 2. Patients and Methods

A total of 394 women were included in this prospective cohort study. Inclusion criteria were singleton pregnancies with one or more of the following risk factors for the development of preeclampsia: Chronic hypertension, SLE and/or APS, maternal thrombophilia, history of preeclampsia, and a pathologic first trimester screening for preeclampsia. Controls were women without any known predisposing risk factors for the development of preeclampsia.

Exclusion criteria were fetal abnormalities, fetal loss before 23 + 0 weeks of gestation, and multiple pregnancies.

Women were recruited at time of admission for prenatal care, starting between 10 and 12 weeks of gestation.

Women with APS fulfilled at least one of the Sydney clinical criteria [24] and women with SLE showed at least 4 of the 11 American College of Rheumatology (ACR) criteria [25]. Apart from APS, maternal thrombophilia was defined as an inherited or acquired condition which predisposes an individual to thromboembolism, such as antithrombin deficiency, APC resistance, Factor V Leiden mutation, Factor II G202I0A, or combined effects [12].

Chronic hypertension, preeclampsia, and HELLP (hemolysis elevated liver enzymes low platelets) syndrome were defined according to international criteria [26]. IUGR (intrauterine growth restriction) was defined as fetal growth <5th percentile of gestational age.

The first trimester screening for preeclampsia consisted of a combination of maternal demographic characteristics, including medical and obstetric history, uterine artery pulsatility index (PI), mean arterial pressure (MAP) and maternal serum pregnancy-associated plasma protein-A (PAPP-A), and placental growth factor (PlGF) at 11–13 weeks gestation [27].

Blood samples were collected without anticoagulant every 4 weeks from time of study inclusion until delivery. Samples were centrifuged at 800× *g* for 10 min; sera were portioned in 200 μL aliquots and stored at the Biobank Graz, Austria, at −80 °C.

sFlt-1 and PlGF were measured using an automated ELISA (Roche Diagnostics GmbH; Mannheim, Germany) according to the manufacturer’s protocol. The detection limit was 6 pg/mL for sFlt-1 and <2 pg/mL for PlGF. The intra-assay coefficients of variation were <2% for sFlt-1 and PlGF, and the inter-assay coefficients of variation were 2.3% to 4.3% for the sFlt-1 assay and 2.7% to 4.1% for the PlGF assay.

The study protocol was approved by the Medical University Ethics Committee (IRB00002556) and all participants gave written informed consent.

### Statistical Analysis

After data closure, all variables passed a plausibility check to detect outliers in the data set. No extreme values have been extracted from the full data set. Assumption of normal distribution was proven with Shapiro–Wilk and Kolmogorov–Smirnov tests (*p* > 0.05 normally distributed data assumed) and Q–Q plots. Comparisons among different outcome groups were tested with Mann–Whitney U and Kruskal–Wallis tests with post-hoc Bonferroni correction for multiple testing. To investigate longitudinal changes over time on different outcome variables and to deal with random effects and unequal sample sizes for the different gestational age weeks (measured for sFlt-1/PlGF-ratio) linear mixed effects models were performed. The linear mixed effects models were performed as restricted maximum likelihood (REML) approach combined with the Satterthwaite’s method. The patient ID of pregnant women was considered as a person-specific random effect. In the intervals for different stages of gestational age, when more than one sample existed per woman, the latest sample was used.

Data are presented as total number, as mean ± standard deviation, or in case of a skewed distribution, as median and interquartile range (25-percentile and 75-percentile). A two-tailed *p*-value of less than *p* < 0.05 was considered as statistically significant. All statistical tests were performed using SPSS version 25.0 (SPSS Inc., Chicago, IL, USA), R version 3.4.1 (package lmer), and GraphPad Prism version 6.05 (GraphPad Software, San Diego, USA) for visualizations.

## 3. Results

The study group consisted of 89 women with chronic hypertension, 44 women with SLE and/or APS, 22 women with thrombophilia, 118 women with a history of preeclampsia, 53 women with a pathologic first trimester screening for preeclampsia, and 68 controls.

Maternal thrombophilia—apart from APS—consisted in most cases of APC resistance, Faktor V Leiden mutation.

Overall, first trimester screening for preeclampsia has been performed in 243 women; 113 women were screened positive for either early or late onset preeclampsia.

Demographic and clinical characteristics are shown in Table 1.

Women with chronic hypertension had a significantly higher prepregnancy BMI and a higher systolic and diastolic blood pressure at study entry (Table 1).

There were no significant differences in maternal age, prepregnancy BMI, and systolic and diastolic blood pressure at study entry between women with APS/SLE, maternal thrombophilia, preeclampsia in a previous pregnancy, pathologic first trimester screening for preeclampsia, and controls (Table 1).

Early onset preeclampsia occurred in three women with chronic hypertension, in two women with APS/SLE, in one woman with thrombophilia (APC-resistance), and in seven women with preeclampsia in a previous pregnancy, with HELLP syndrome complicating two of them. One woman with a pathologic first trimester screening for preeclampsia developed early onset preeclampsia and one woman of the control group.

Late onset preeclampsia occurred in thirteen women with chronic hypertension, in six women with APS/SLE, and in seven women with preeclampsia in a previous pregnancy. Six women with a pathologic first trimester screening for preeclampsia developed late onset preeclampsia and three women of the control group.

IUGR was present in ten pregnancies; two in women with chronic hypertension, preeclampsia in a previous pregnancy, and in controls, respectively, as well as in four women with APS/SLE.

Seventy women (78.6%) with chronic hypertension received LDA (25.7% 75 mg; 37.1% 100 mg; 37.1% 150 mg), starting at first trimester, in 30 cases (42.9%) due to a pathologic first trimester screening for preeclampsia (Table 1). From the beginning of pregnancy, 40 women (90.9%) with APS/SLE received LDA (10% 75 mg; 57.5% 100 mg; 32.5% 150mg). Women (84.1%) with APS/SLE received low-molecular-weight heparin (LMWH, enoxaparin) from diagnosis of pregnancy until 6 weeks postpartum (Table 1). Receiving LMWH from the beginning of pregnancy until 6 weeks postpartum were 21 women with APS/SLE (95.5%), and 10 women (45.5%) received LDA (10% 75 mg; 50% 100 mg; 40% 150 mg), starting at first trimester, in two cases (20%) due to a pathologic first trimester screening for preeclampsia. One hundred and ten (93.2 %) women with a history of preeclampsia received LDA (11% 75 mg; 54.7% 100 mg; 31.9% 150 mg) from the beginning of pregnancy (Table 1). After a few weeks, eight women stopped taking LDA self-reliant. Fifty-one women (96.2%) with a pathologic first trimester screening for preeclampsia received LDA (25.7% 75 mg; 50.4% 100 mg; 23.9% 150 mg). None of the women of the control group received LDA nor LMWH. Despite treatment with LDA, 22 women developed preeclampsia; only six of them developed preeclampsia before 34 weeks of gestation.

### 3.1. Longitudinal Changes of Sflt-1 /Plgf Ratio during Pregnancy in Women with Adverse Obstetric Outcome Compared to Women with Normal Pregnancies

**Figure 1:**
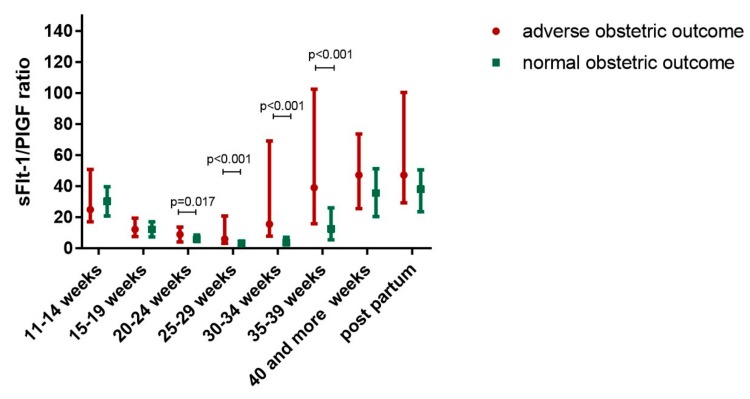
Longitudinal changes of soluble FMS-like Tyrosine Kinase 1 (sFlt-1)/placental growth factor (PlGF) ratio during pregnancy in women with adverse obstetric outcome compared to women with normal pregnancies.

The sFlt-1/PlGF ratio was significantly higher in women with an adverse obstetric outcome compared to women with a normal pregnancy in all six study groups, starting between 20 and 24 weeks of gestation (Table 2). The sFlt-1/PlGF ratio increased with gestational age in both women with an adverse obstetric outcome and women with normal pregnancies, but to a much higher extent in women with adverse obstetric outcome (*p* < 0.001) (Figure 1, Table 2 and Table 3).

### 3.2. Effect of Lda on Sflt-1/Plgf Ratio in Women with and without Adverse Obstetric Outcome

There was no effect of LDA on sFlt-1/PlGF ratio in women with and without adverse obstetric outcome (Table 4).

Additionally, there was no effect of LDA on sFlt-1/PlGF ratio, regardless obstetric outcome (Table 5).

### 3.3. Effect of Lda on Sflt-1/Plgf Ratio in Different Study Groups

**Figure 2:**
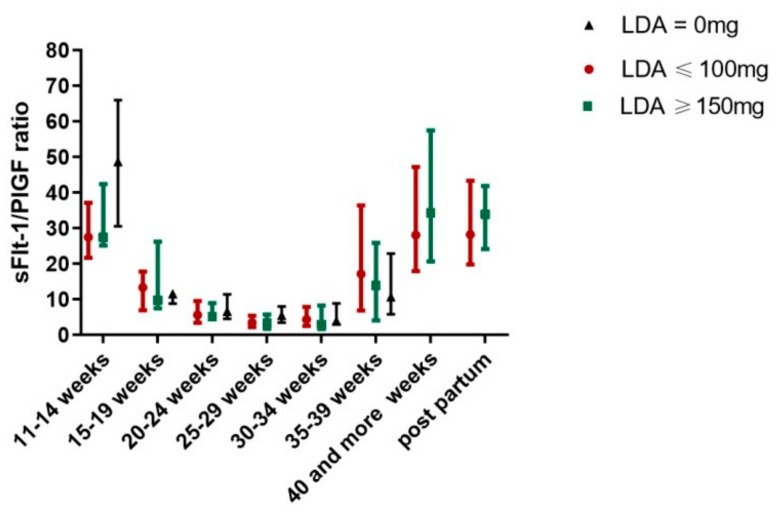
Effect of LDA on sFlt-1/PlGF ratio in women with a pathologic first trimester screening for preeclampsia.

LDA treatment affected the sFlt-1/PlGF ratio in women with a pathologic first trimester screening for preeclampsia (group 6) in a dose-dependent manner (Figure 2). The use of LDA showed a trend towards an improved sFlt-1/PlGF ratio in women with preeclampsia in a previous pregnancy (group 4), but results did not reach significance.

There was no effect of LDA on sFlt-1/PlGF ratio in women with chronic hypertension (group 1), in women with APS/SLE (group 2), in women with thrombophilia (group 3) and controls (group 5) (Table 6).

## 4. Discussion

The main finding of this prospective longitudinal study showed that the use of LDA affected the sFlt-1/PlGF ratio in women with a pathologic first trimester screening for preeclampsia in a dose-dependent manner and showed a trend towards an improved sFlt-1/PlGF ratio in women with preeclampsia in a previous pregnancy.

Recent studies have suggested that aspirin and aspirin-like compounds have a variety of actions in addition to their well-studied ability to inhibit cyclooxygenases. Some of these effects may act via different pathways from those that enhance the trophoblast [26].

The effect of aspirin on angiogenic factors, such as sFlt-1 and PlGF is the subject of ongoing interest and has been examined in several in vitro studies [27,28,29,30,31].

Li et al. reported that aspirin seems to be able to block the production of sFlt1 in the placenta in a dose-dependent manner, suggesting that aspirin exerts its therapeutic effects via cyclooxygenase-1 inhibition [27]. However, Xu et al. demonstrated in their in vitro study that aspirin improves trophoblast cell integration by inhibiting the effect of TNF-alpha via PGI2, but without affecting VEGF, PlGF and sFlt-1 [28].

Panagodage et al. revealed that aspirin modulates the production of cytokines and improves trophoblast function; additionally, it increases the secretion of PlGF from the trophoblast [29].

Su et al. investigated the effect of aspirin on trophoblast cell function and its effect on sFlt-1. The authors demonstrated that aspirin enhances cell invasiveness and inhibits sFlt-1 production in trophoblasts. Moreover, sFlt-1 itself also inhibits trophoblast invasion [30].

However, the underlying pathophysiology of how aspirin prevents preeclampsia is not fully understood. The interaction of aspirin, preeclampsia and angiogenic factors such as sFlt-1 and PlGF, the role with the disease process and if aspirin has, if any, an effect on angiogenic factors is still unknown.

Mone et al. aimed to determine the impact of low-dose aspirin in low-risk pregnancies on several biomarkers such as PAPP-A and PlGF as well as on maternal blood pressure, fetal growth parameters, and histological findings of the placenta [31]. The authors could not find any significant impact of low-dose aspirin on these parameters. However, only 75 mg of aspirin was prescribed; it might be speculated that the results may be different with higher dosages.

According to our results, LDA seems to have an effect on the sFlt-1/PlGF ratio women with a pathologic first trimester screening for preeclampsia.

Murtoniemi et al. aimed to study the effect of LDA 100 mg on maternal PlGF concentrations in women with clinical risk factors for preeclampsia and of low-risk women and revealed an association of LDA and a higher increase in serum PlGF concentration in women at high risk for preeclampsia during pregnancy [32]. Our results confirm these findings and even show a dose-dependent effect of aspirin with the most prominent effects at a dosage of 150 mg.

We were not able to detect the same effect in women with chronic hypertension, APS/SLE, thrombophilia, as well as in women with preeclampsia in a previous pregnancy; it might be speculated that the effectiveness of aspirin differs among various entities.

The sFlt-1/PlGF ratio was significantly higher in women with an adverse obstetric outcome compared to women with a normal pregnancy in high- and low-risk pregnancies and showed an increasing trend throughout gestation

Several studies already reported altered maternal concentrations of sFlt-1 and PlGF before and at the onset of preeclampsia [33,34,35,36,37,38,39,40,41] and therefore have a high predictive value [42,43,44,45,46,47,48,49,50]. Our results strongly support these finding.

Khalil et al. investigated longitudinal changes of maternal serum concentrations of sFlt-1 and PlGF in 243 women with a pathologic first trimester screening for preeclampsia [49]. The authors demonstrated a higher predictive value of repeated measurements of angiogenic factors compared to single measurements. However, they could not find any significant association of sFlt-1 and PlGF-levels and several maternal characteristics such as history of preeclampsia or chronic hypertension. These findings are in line with our results, as we were not able to reveal any significant differences of the sFlt-1/PlGF ratio between women with chronic hypertension or a history of preeclampsia, as well as women with APS/SLE, thrombophilia, a pathologic first trimester screening for preeclampsia and controls.

Powers et al. aimed to reveal differences of angiogenic factors in 993 high-risk pregnancies throughout gestation, consisting of women with pre-existing diabetes, chronic hypertension, and preeclampsia in a previous pregnancy as well as multi-fetal pregnancies. The authors reported significantly higher levels of sFlt-1 and endoglin and significantly lower levels of PlGF in women who developed preeclampsia but also noted that these changes are similar to those in low-risk pregnant women. Additionally, they could not find any differences in concentrations of sFlt-1 and PlGF between women with or without aspirin prophylaxis treatment, suggesting that aspirin does not have a significant effect on the concentration of angiogenic factors [50].

## 5. Conclusions

We were able to reveal an association of LDA and sFlt-1/PlGF ratio in women with a pathologic first trimester screening for preeclampsia and showed a dose-dependent effect of aspirin with the most prominent effects at a dosage of 150 mg.

However, larger sample sizes are needed for a distinct interpretation and to confirm a specific trend of the statistical analysis.

Additionally, we demonstrated that the sFlt-1/PlGF ratio was significantly higher in women with an adverse obstetric outcome compared to women with a normal pregnancy in high- and low- risk pregnancies and showed an increasing trend throughout gestation.

The strengths of the present study are the prospective design of the study, as well as the longitudinal measurements of angiogenic factors every 4 weeks, the comparison of angiogenic factors in women with different risk factors for the development of preeclampsia, as well as the determination of an effect of LDA on angiogenic factors.

A limitation of the study is the rather small sample size of individual study groups.

## Figures and Tables

**Table 1:** Demographic characteristics of women included in the study.

	Chronic Hypertension (n = 89)	APS/SLE (n = 44)	Thrombophilia (n = 22)	History of Preeclampsia (n = 118)	Pathologic First Trimester Screening (n = 53)	Controls (n = 68)	P
**Maternal age (years)**	33.4 ± 5.8	32.4 ± 6.2	29.45 ± 5.9	32.9 ± 4.5	33.9 ± 4.8	34.1 ± 6	n.s.
**Prepregnancy BMI (kg/m²)**	31.02 ± 7.4	23.9 ± 4.5	26.4 ± 6.7	25.5 ± 5.6	25.5 ± 6.5	26.5 ± 6.7	0.001
**Average systolic blood pressure (mm Hg)**	143.8 ± 19.2	132.6 ± 11.2	129.8 ± 16.2	135.8 ± 12.8	131.2 ± 13.2	128.9 ± 15.1	0.001
**Average diastolic blood pressure (mm Hg)**	93.1 ± 13.1	84.2 ± 8.2	85.5 ± 15.9	91.5 ± 12.8	89.5 ± 10.8	79.1 ± 11.7	0.001
**Ethnicity**							
African, no. (%)	2 (2.2%)	1 (2.2%)	0	0	0	0	
Arabian, no. (%)	2 (2.2%)	2 (4.5%)	0	4 (3.4%)	0	0	
Asian, no. (%)	0	0	0	1 (0.8%)	0	0	
Caucasian, no. (%)	85 (95.5%)	41 (93.3%)	22 (100%)	113 (95.8%)	53 (100%)	68	
**low dose aspirin**							
Overall, no. (%)	70 (78.6%)	40 (90.9%)	10 (45.5%)	110 (93.2%)	51 (96.2%)	0	
<100 mg, no. (%)	18 (20.2%)	4 (9.1%)	1 (4.5%)	13 (11%)	7 (13.2%)	0	
100 mg, no. (%)	26 (29.2%)	23 (52.2%)	5 (22.7%)	60 (50.8%)	29 (54.7%)	0	
150 mg, no. (%)	26 (29.2%)	13 (29.5%)	4 (18.2%)	37 (31.45)	15 (12.7%)	0	
**preeclampsia**	16 (17.9%)	8 (18.2%)	1 (4.5%)	19 (16.15)	6 (11.3%)	4 (5.9%)	
**Gestational age at delivery**	38.9 ± 1.1	37.8 ± 3	39.1 ± 3.3	38.8 ± 2	40.1 ± 1.1	39.5 ± 1.8	n.s.
**Mode of delivery**							
Spontaneous delivery, no. (%)	30 (33.7%)	12 (27.3%)	10 (45.5%)	48 (40.7%)	23 (43.4%)	36 (52.9%)	
caesarean section, no. (%)	56 (62.9%)	28 (63.6%)	11 (50%)	69 (58.5%)	23 (43.4%)	28 (41.2%)	
vaginal assisted delivery, no. (%)	3 (3.4%)	4 (9.1%)	1 (4.5%)	1 (0.8%)	7 (13.2%)	4 (5.9%)	

**Table 2:** Longitudinal changes of sFlt-1 /PlGF ratio during pregnancy in women with adverse obstetric outcome compared to women with normal pregnancies.

	Adverse Obstetric Outcome (n = 54)	Normal Obstetric Outcome (n = 339)	P	P Adj
**sFlt-1/PlGF ratio 11–14 weeks**	24.92 (17.06–50.79)	30.26 (20.85–39.64)	0.450	1.000
**sFlt-1/PlGF ratio 15–19 weeks**	12.23 (7.53–19.42)	12.14 (7.35–17.12)	0.739	1.000
**sFlt-1/PlGF ratio 20–24 weeks**	8.95 (4.09–13.69)	5.67 (3.69–8.56)	0.002	0.017
**sFlt-1/PlGF ratio 25–29 weeks**	6.00 (3.20–20.84)	3.02 (1.85–4.73)	0.000	*p* < 0.001
**sFlt-1/PlGF ratio 30–34 weeks**	15.53 (7.78–69.13)	3.66 (2.22–7.03)	0.000	*p* < 0.001
**sFlt-1/PlGF ratio 35–39 weeks**	39.05 (15.82–102.61)	12.48 (15.39–26.09)	0.000	*p* < 0.001
**sFlt-1/PlGF ratio ≥ 40 weeks**	47.23 (25.58–73.68)	35.74 (20.50–51.29)	0.172	1.000
**sFlt-1/PlGF ratio post-partum**	47.24 (29.34–100.39)	38.02 (23.50–50.51)	0.115	0.917

**Table 3:** Longitudinal changes of sFlt-1 /PlGF ratio during pregnancy.

Parameter	sFlt/PlGF Ratio		
**Fixed Part**	Estimate	SE	P
**Intercept**	9.51	1.81	*p* < 0.001
**outcome**	3.14	4.91	0.52
**visits**	1.65	0.36	*p* < 0.001
outcome*visits	5.89	1.04	*p* < 0.001

**Table 4:** Effect of low-dose aspirin (LDA) on sFlt-1/PlGF ratio in women with adverse obstetric outcome.

	Yes (n = 44)	No (n = 10)	P	P Adj
**sFlt-1/PlGF ratio 11–14 weeks**	25.46 (18.94–49.49)	17.23 (15.24–84.12)	0.484	1.000
**sFlt-1/PlGF ratio 15–19 weeks**	11.80 (7.39–21.31)	15.45 (15.45–15.45)	n.a.	n.a.
**sFlt-1/PlGF ratio 20–24 weeks**	8.22 (3.90–12.22)	11.95 (8.06–23.31)	0.079	0.630
**sFlt-1/PlGF ratio 25–29 weeks**	5.52 (3.05–19.96)	16.43 (8.53–25.73)	0.171	1.000
**sFlt-1/PlGF ratio 30–34 weeks**	14.39 (7.16–106.19)	22.54 (8.16–59.23)	0.902	1.000
**sFlt-1/PlGF ratio 35–39 weeks**	37.49 (16.67–102.25)	41.40 (12.53–116.06)	0.859	1.000
**sFlt-1/PlGF ratio ≥ 40 weeks**	43.16 (17.05–74.10)	47.24 (33.99–158.25)	0.558	1.000
**sFlt-1/PlGF ratio post-partum**	56.42 (29.34–100.39)	46.41 (28.19–194.60)	0.806	1.000

**Table 5:** Longitudinal changes of sFlt-1 /PlGF ratio during pregnancy.

Parameter	sFlt/PlGF Ratio		
Fixed Part	Estimate	SE	P
**Intercept**	12.12	3.13	0.000
**ASS**	−1.99	3.79	0.599
**visits**	2.50	0.60	*p* < 0.001
ASS*visits	−0.39	0.74	0.593
SE=standard error			

**Table 6:** Longitudinal changes of sFlt-1 /PlGF ratio during pregnancy.

Parameter	sFlt/PlGF Ratio		
Fixed Part	Estimate	SE	P
**chronic hypertension**			
**Intercept**	2.00	10.23	0.845
**LDA**	3.20	11.36	0.778
**visits**	3.73	2.05	0.070
LDA *visits	−0.17	2.29	0.940
**APS/SLE**			
**Intercept**	7.03	12.22	0.566
**LDA**	5.83	12.75	0.648
**visits**	1.00	2.35	0.672
LDA *visits	−0.26	2.46	0.915
**thrombophilia**			
**Intercept**	26.80	16.99	0.130
**LDA**	−12.65	25.34	0.623
**visits**	1.93	1.37	0.164
LDA*visits	−2.05	1.95	0.297
**history of adverse obstetric outcome**			
**Intercept**	11.22	12.10	0.354
**LDA**	2.82	12.55	0.822
**visits**	3.14	2.08	0.132
LDA*visits	−1.97	2.18	0.368
**pathologic first trimester screening**			
**Intercept**	42.79	18.94	0.025
**LDA**	−39.47	19.66	0.046
**visits**	−6.73	4.25	0.115
LDA*visits	10.78	4.36	0.015
SE=standard error			
